# *Cutibacterium avidum* resists surgical skin antisepsis in the groin—a potential risk factor for periprosthetic joint infection: a quality control study

**DOI:** 10.1186/s13756-021-00883-1

**Published:** 2021-02-01

**Authors:** Steven M. Maurer, Laura Kursawe, Stefan Rahm, Julia Prinz, Annelies S. Zinkernagel, Annette Moter, Stefan P. Kuster, Reinhard Zbinden, Patrick O. Zingg, Yvonne Achermann

**Affiliations:** 1 Division of Infectious Diseases and Hospital Epidemiology, University Hospital Zurich, University of Zurich, Zurich, Switzerland; 2 Institute of Medical Microbiology, University of Zurich, Zurich, Switzerland; 3 Department of Orthopedics, University Hospital Balgrist, Zurich, Switzerland; 4 Institute of Microbiology, Infectious Diseases and Immunology, Charité-Universitätsmedizin Berlin, Berlin, Germany; 5 Department of Dermatology, University Hospital Zurich, University of Zurich, Zurich, Switzerland

**Keywords:** *Cutibacterium avidum*, *Cutibacterium* species, Skin antisepsis, Periprosthetic joint infection, Hip

## Abstract

**Background:**

The skin commensal *Cutibacterium avidum* has been recognized as an emerging pathogen for periprosthetic joint infections (PJI). One currently assumes that the early occurring PJIs are a consequence of skin commensals contaminating the peri-implant tissue during surgery. We addressed whether standard skin antisepsis with povidone-iodine/alcohol before total hip arthroplasty (THA) is effective to eliminate colonizing bacteria with focus on *C. avidum*.

**Methods:**

In a single-center, prospective study, we screened all patients for skin colonizing *C. avidum* in the groin before THA. Only in the patients positive for *C. avidum*, we preoperatively repeated skin swabs after the first and third skin antisepsis and antibiotic prophylaxis. We also obtained dermis biopsies for microbiology and fluorescence in situ hybridization (FISH).

**Results:**

Fifty-one out of 60 patients (85%) were colonized on the skin with various bacteria, in particular with *C. avidum* in 12 out of 60. Skin antisepsis eliminated *C. avidum* in eight of ten (20%) colonized patients undergoing THA. Deeper skin (dermis) biopsies were all culture negative, but FISH detected single positive ribosome-rich *C. avidum* in one case near sweat glands.

**Conclusion:**

Standard skin antisepsis was not effective to completely eliminate colonizing *C. avidum* on the skin in the groin of patients undergoing THA. Colonizing with *C. avidum* might pose an increased risk for PJI when considering a THA. Novel more effective antisepsis strategies are needed.

*Trial registration* No clinical trial

## Background

Absolute numbers of periprosthetic joint infections (PJI) are increasing due to the increasing aging population with the need of joint prostheses [1]. Most commonly isolated microorganisms are staphylococci, streptococci, enterococci, gram-negative bacteria, and facultative anaerobic bacteria such as *Cutibacterium* species (mainly *Cutibacterium acnes and Cutibacterium avidum* [formerly *Propionibacterium acnes and avidum, respectively*]) [2]. These bacteria cause infections, which are difficult to treat because they hide in a self-made biofilm consisting of an extracellular matrix of polymeric substances [3]. Notably, most of the currently available antibiotics are not active against bacteria in biofilms due to the bacterial persistence and tolerance [4, 5]. Treatment of PJI requires aggressive surgical debridement with prolonged usage of antibiotics or even the exchange of the whole prosthesis [2]. Most of the PJI occur when viable bacteria on the skin surface or dermis contaminate deeper tissue layers and eventually the arthroplasty at the time of surgery [6–8]. Therefore, pre- or intraoperative prevention strategies are key to avoid intraoperative bacterial contamination. Prevention strategies are multifaceted with the focus on perioperative antibiotic prophylaxis within 30–60 minutes before surgery and on skin antisepsis immediately before surgical incision of the skin [7, 9, 10]. Various skin antisepsis agents are in use, among others povidone-iodine (PVI), chlorhexidine gluconate (CHG), or alcohol [7], which target the natural inhabitants of the human skin [11, 12].

*C. avidum* was recently identified as an emerging pathogen in hip arthroplasty infections [13–15]. In a prospective study, we found that 32.3% of all patients undergoing hip arthroplasty surgery were colonized with *C. avidum* in the groin region, which is rich of sweat glands [16]. Obesity with more sweating was a predisposing factor for colonization in those patients [16] and thus the likely explanation for the increasing number of *C. avidum* PJI in our center [17]. *Cutibacterium* species are also found in deeper skin, near sebaceous or sweat glands. This propensity may render them more resistant to antiseptic agents*.* Therefore, we investigated if the standard skin antisepsis we use in our clinic eliminates efficiently all *C. avidum* skin colonization at time of surgical incision by swab cultures of superficial and deeper skin structures as well as by FISH in the dermis.

## Methods

### Study design and patient population

In a single-center prospective study at the University Hospital Balgrist in Zurich, Switzerland, we screened patients 8–14 days prior to hip surgery for *C. avidum* colonization and tested the intraoperative effect of skin antisepsis on *C. avidum*. We included all patients with a planned primary THA surgery through a direct anterior approach, i.e. between musculus tensor fascia latae and musculus sartorius [18] from October 2018 until April 2019. Basic clinical characteristics, i.e. age, sex, BMI, type of operation of the patients were retrieved from the charts. In the patients screened positive for *C. avidum,* we intraoperatively searched for viable bacteria of superficial and deeper skin structures using routine culture techniques and FISH after triple skin antisepsis.

In every patient antibiotic prophylaxis with single-dose cefuroxim (1.5 g in patients < 80 kg and 3 g in patients ≥ 80 kg) was administered 30–60 min prior to THA. No additional preoperative decolonization or antiseptic showering was performed on the day before surgery.

### Skin antisepsis, skin swab and dermis biopsies

For antisepsis we used Betaseptic® solution (Mundipharma, Limburg, Germany) which contains 3.24 g povidone-iodine, 38.9 g 2-propanol and 38.9 g ethanol 96% in 100 ml solution according to the manufacturer. If the patient had an allergy against iodine, antisepsis was performed with Kodan® (Schülke, Norderstedt, Germany), which is a 2-propanol/1-propanol/biphenyl-2-ol solution containing 45.9 g 2-Propanol, 10.0 g 1-Propanol, 0.20 g Biphenyl-2-ol, 30% hydrogen peroxide and purified water in 100 ml solution, according to the manufacturer. Surgical antisepsis was performed 3 times for one minute with a one-minute interval in between. Due to the time required for collection of skin scrapings after first and third antisepsis, total duration of the antisepsis procedure was up to 5 min before incision of the skin. The sterile blades were changed after the first and second scraping procedure to prevent cross-contamination.

Skin swabs were taken by scraping over the skin in the groin near the planned anterior incision with the same sterile blade 4 to 5 times. The skin material on the blade was collected with eSwabs (Copan, Brescia, Italy).

Immediately after surgical skin incision for THA implantation, one biopsy from the dermis at the edge of surgical anterior incision was obtained (5 cm in length, 0.3–0.5 cm in width and 0.5 cm in depth) and cut into two pieces, one was used for evaluation of bacterial species and one for FISH.

### Microbiological analysis of skin swabs and dermis biopsies

eSwabs and dermis biopsies were analyzed for bacterial growth at the Institute of Medical Microbiology of the University of Zurich. For aerobe cultivation, swabs were streaked out onto Columbia sheep blood agar without antibiotics (bioMérieux; Mary-l’Etoile, France) and onto colistin-nalidixic acid blood agar (bioMérieux). A *Brucella* agar plate (in-house 10% sheep blood agar [Becton Dickinson] with hemin and vitamin K1 [Sigma-Aldrich Merck]) was inoculated for anaerobe cultivation. All aerobic plates were incubated for 7 days at 37 °C. The anaerobe environment was ensured with GEN bags (bioMérieux). Focusing on anaerobe growing bacteria, skin swabs after skin antisepsis were solely anaerobically incubated on *Brucella* agar plates for 10 days at 37 °C. Skin biopsies were processed in the same way as skin swabs except an additional thioglycolate broth (bioMérieux) was incubated for 10 days and a MacConkey-Agar (bioMérieux), a Chocolate agar PolyViteX (bioMérieux) and a PEA agar plate (bioMérieux) were incubated for 2 days. All bacteria were identified by matrix-assisted laser desorption ionization (MALDI-TOF) using a Bruker MALDI Biotyper (Becton Dickinson). We used the three-phase streaking pattern for a semiquantitative analysis for *C. avidum* in all skin swab samples. We defined low level of bacteria when growth was observed in the first section (+), medium level when growth in the second section (++), and high level when growth in the third and last section (+++).

### Visualization of bacteria of dermis biopsies with FISH

To visualize the presence of bacteria below the skin surface in the dermis, we performed FISH of intraoperatively acquired biopsies at the Biofilm center in Berlin, Germany. To immediately fix the dermis samples in the operating theatre, we used a FISH-fixation solution, optimized for the detection of a wide variety of microorganisms including gram-positive, gram-negative bacteria and fungi (FISHopt ®, MoKi Analytics Germany) [19].

2 µm sections of approximately 1 cm dermis samples were prepared and analyzed by FISH as previously described [20, 21]. Briefly, the fixed samples were embedded in cold polymerizing resin Technovit 8100 (Kulzer, Wehrheim, Germany) according to the manufacture`s manual and cut in 2 µm thick sections. Cross sections of ten dermis biopsies were embedded and sectioned including epidermis, dermis and subcutaneous tissue per section. At least four sections per sample were analyzed using FISH. For the hybridization process, a preheated hybridization buffer was added to the sample which contained fluorescently labeled probes corresponding to the target rRNA. The whole tissue sections were analyzed microscopically by two independent investigators for approximately 45 min each.

We used the pan-bacterial probe (EUB338) and the specific FISH probe (PAC) [22–24], which manly targets *C. acnes* but shows also 100% homology to closely related *Cutibacterium modestum* (formerly *C.utibacterium humerusii*), which was originally identified from a joint infection [25], *Cutibacterium namnetense*, *Acidithiobacillus thiooxidans*, *Mycobacterium chitae* (Silva Database, 24. November. 2020, selection, Additional file 1: Table S3). To visualize *C. avidum,* we designed and evaluated a specific probe (PRAV) (Table 1, Additional file 1: Table S2, Figure S1, Figure S2). In silico evaluation of the FISH probe PRAV was accomplished using the software probeCheck (http://131.130.66.200/cgi-bin/probecheck/content.pl?id=home), which revealed 100% homology with *C. avidum* and four closely related species *Acidipropionibacterium (*formerly *Propionibacterium) acidipropionici, Acidipropionibacterium (formerly Propionibacterium) propionicum, Acidipropionibacterium (formerly Propionibacterium) jensenii, and Acidipropionibacterium (formerly Propionibacterium) thoenii* [26] (Additional file 1: Table S2). Except for *C. avidum,* these species have no clinical relevance in PJI. Sensitivity and specificity of the probe PRAV was confirmed using fixed cultures of *C. avidum* MM433 (clinical isolate), *C. avidum* strain (clinical isolate of this study), and *C. acnes* MM1127 (clinical isolate) as described in the supplement (Additional file 1: Table S4). Identity of all strains was confirmed by 16S rRNA-gene sequencing using TPU1 and RTU3 primers as previously describted [27]. Amplicons were sequenced using a commercial sequencing facility (Microsynth AG, Switzerland) and compared to all currently available sequences from the public databases (EMBL and GenBank) and the curated centroid database of the program SmartGene (SmartGene Inc., Switzerland) [28]. In addition, a tissue biopsy of the pseudocapsule of a proven PJI with positive growth of *C. avidum* was used as a positive control.

**Table 1.** Probes used for fluorescence in situ hybridization in dermal biopsies

Probe	Target	Specificity	References
EUB338	16S rRNA	Most bacteria	Amann [48]
PAC	16S rRNA	***Cutibacterium acnes*** *Cutibacterium modestum* *Cutibacterium namnetense* *Acidothiobacterium thiooxidans* *Mycobacterium chitae*	Poppert [23]
*PRAV	16S rRNA	***Cutibacterium avidum*** *Acidipropionibacterium acidipropionici* *Acidipropionibacterium propionicum* *Acidipropionibacterium jensenii* *Acidipropionibacterium theonii*	This study

*Further information regarding the sequences in the Additional file 1: Table S2

The nucleic acid stain 4′,6-diamidino-2-phenylindole (DAPI) was applied to all samples to visualize microorganisms and cell host nuclei. Microscopic evaluation was performed using an epifluorescence microscope (AxioImager z1, Carl Zeiss, Germany) with narrow band filter sets Cy3, Cy5 and FITC (AHF Analysentechnik, Germany). Digital images were taken using the ZEN software delivered with the instrument.

### Statistical methods

Descriptive statistics were performed using Excel version 16.41. The binominal confidence limits in Fig. 2 were calculated using Stata version 14.2 (StataCorp) . Futher statistics were not performed due to a small number of patients.

## Results

From October 2018 to April 2019, we enrolled 60 patients prior to THA in our study. Approximately half of the patients (46.7%; 28) were female with a median age of 67 (range, 40–87 years). The median BMI of all patients was 26.7 kg/m^2^ (range, 19.1–39.1), whereof the 12 patients with positive *C. avidum* colonization had a higher BMI with a median of 32.1 kg/m^2^ (range, 21.8–39.1). Almost all patients (98.3%) underwent primary THA with an anterior approach due to osteoarthritis, except for one patient, who required the operation due to femoral head necrosis. In 55% (33 of 60) of patients, THA was performed on the left hip (Additional file 1: Table S1).

**Fig. 1:**
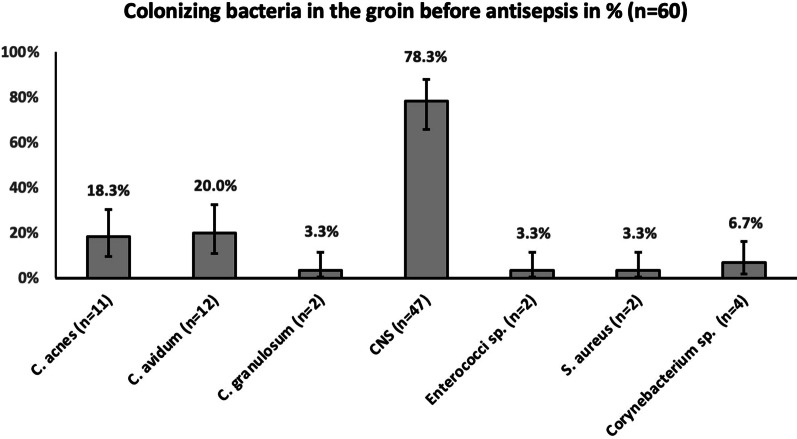
Percentage (with binomial confidence limits) of skin colonizing bacteria in the groin before antiseptic treatment. *C. acnes, Cutibacterium acnes; C. avidum, Cutibacterium avidum; C. granulosum, Cutibacterium granulosum*; *CNS*, *coagulase-negative staphylococci*; *S. aureus, Staphylococcus aureus*; *Corynebacterium* sp. included *C. tuberculostearicum, C. glucuronolyticum, C. singular,* and *C. accolens*; Enterococci sp. included one *E. faecalis* and one *E. faecium*. CNS included *S. epidermidis* in n = 29 (48.3%), *S. hominis* in n = 22 (36.7%), *S. capitis* in n = 8 (13.3%), *S. haemolyticus* in n = 6 (10%), *S. warneri* in n = 3 (5%), *S. pettenkoferi* in n = 2 (3.3%), *S. lugdunensis* in n = 2 (3.3%), *S. simulans* in n = 1 (1.7%)

Only 12 of 60 (20%) patients were colonized with *C. avidum* while most of the patients were colonized with other bacteria such as coagulase-negative staphylococci (CNS) (47, 78.3%), *C. acnes* (11, 18.3%), *Corynebacterium* sp. (4, 6.7%), *Cutibacterium granulosum* (2, 3.3%), *Enterococci* sp*.* (2, 3.3%), *S. aureus* (2, 3.3%) (Fig. 1, Additional file 1: Table S1). Ten out of 12 patients colonized with *C. avidum* were intraoperatively tested for persistent bacterial growth after routine skin antisepsis and perioperative antibiotic prophylaxis. Two *C. avidum*-positive patients were excluded due to change of operation date. We found viable bacteria in eight of ten (80%) after the first and in five of ten (50%) patients after the third round of antisepsis. Focusing on absolute *C. avidum* growth rate, antisepsis was ineffective in four of ten (40%) after the first and two of ten (20%) after the third antisepsis. The semiquantitative analysis of *C. avidum* before and after antisepsis generally shows low amount of bacterial growth (Table 2). In contrast, dermis biopsies were in all cases culture negative.

**Table 2:** Bacterial growth results after skin antisepsis of 10 patients colonized with *C. avidum* at time of hip arthroplasty surgery

Patient-Nr.	Preoperative skin swabs (outpatient clinic) within 1–2 weeks before surgery	Perioperative skin swabs	
		After first antisepsis	After third antisepsis
4	***C. avidum (+)***	No growth	No growth
	*S. epidermidis*		
17	***C. avidum (+)***	***C. avidum (+)***	***C. avidum (+)***
23	*C. granulosum*	No growth	*C. granulosum*
	***C. avidum (++)***		
	*S. epidermidis*		
	*S. haemolyticus*		
	*S. epidermidis*		
24	***C. avidum (++)***	***C. avidum (+)***	No growth
	*S. epidermidis*	*S. epidermidis*	
	*C. acnes*	*C. acnes*	
		*C. granulosum*	
32	***C. avidum (+)***	*S. capitis*	No growth
	*S. epidermidis*		
	*S. hominis*		
34	***C. avidum (+)***	*C. acnes*	No growth
	*S. epidermidis*		
	*Staphylococcus lugdunensis*		
	*Staphylococcus capitis*		
	*Corynebacterium tuberculostearicum*		
43	***C. avidum (+)***	*S. epidermidis* *S. hominis* *S. haemolyticus* *S. capitis*	*S. epidermidis*
45	***C. avidum (+)*** *Enterococcus faecalis* *Enterococcus facium* *S. epidermidis*	***C. avidum (+)***	No growth
51	***C. avidum (+)*** *S. epidermidis*	***C. avidum (++)***	***C. avidum (+)*** *S. epidermidis*
53	*S. epidermidis* ***C. avidum (+)*** *S. lugdunensis*	*S. epidermidis* *C. acnes* *S. hominis*	*S. epidermidis*

We used the three-phase streaking pattern for a semiquantitative analysis for *C. avidum* in all skin eSwab samples. We defined low level of bacteria when growth in the first section (+), medium when growth in the second section (++), and high when growth in the third and last section (+++)

FISH was applied in all ten culture-negative dermis biopsies. FISH is a molecular technique with fluorescently labeled oligonucleotide probes which target in our case specifically ribosomes. Ribosomes are highly abundant in actively replicating bacterial cells. The FISH signal intensity correlates with the ribosomal content and therefore can be used as activity marker [29–32]. However, since the ribosome content may vary between bacteria, the absence of a FISH signal cannot differentiate between a dead or resting state. To counterstain all bacteria, also the ones not detected by FISH, we used the nucleic acid specific stain DAPI, which detects host cell nuclei as well as microorganisms.

**Fig. 2:**
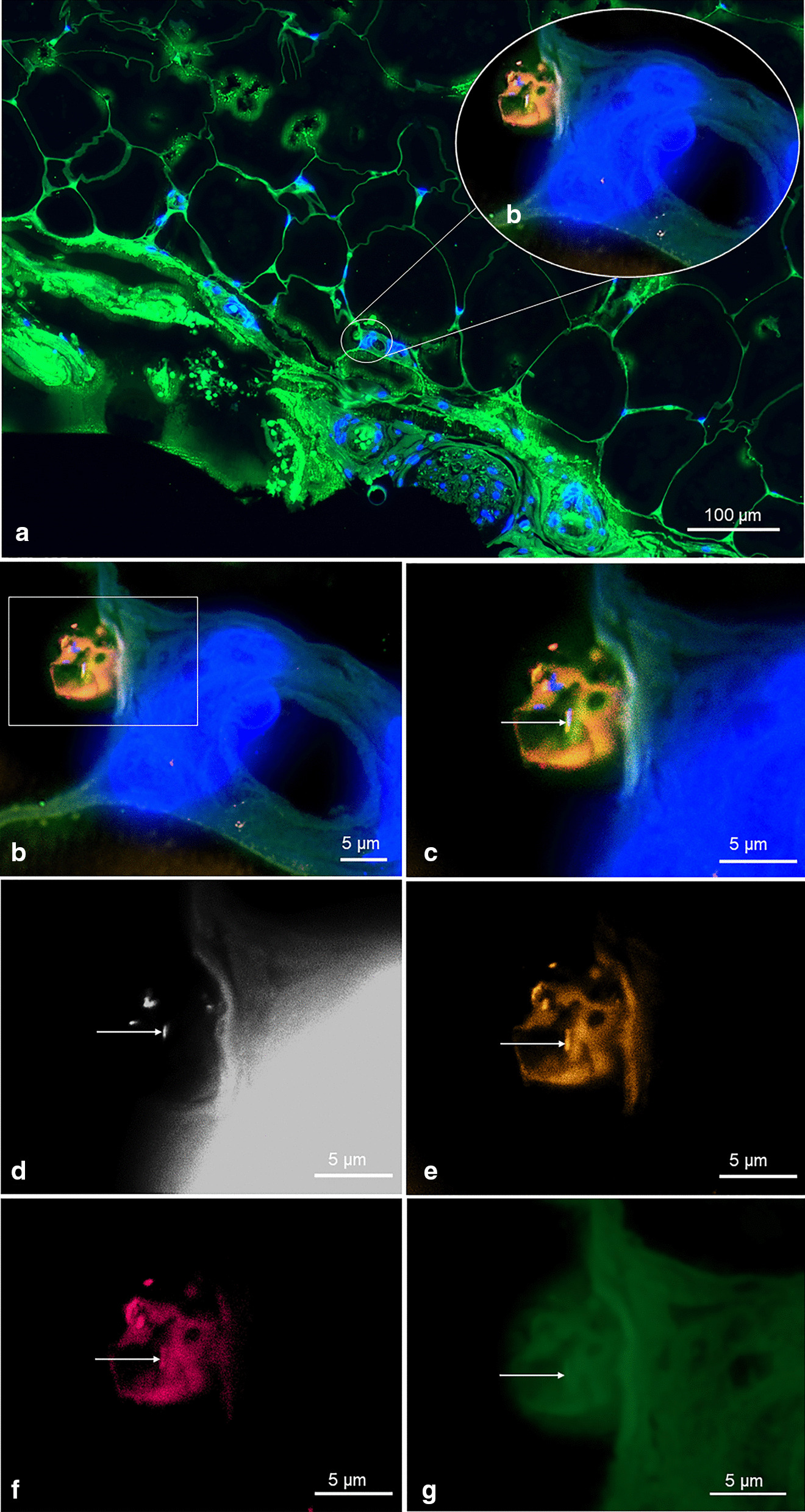
FISH of a dermis biopsy shows single *C. avidum* cells located close to structures compatible with sweat glands, located at the border of the dermis to the subcutaneous fat tissue. The tissue section was hybridized with the panbacterial probe EUB338_Cy5_ (mangenta), the *C. acnes/ C. modestum/ C. namnetense/ A. thiooxidans/ M. chitae*-specific probe PAC_FITC_ (green) and the *C. avidum/ A. thiooxidans/ A. propionicum/ A. jensenii/ A. thoeni*–specific probe PRAV_Cy3_ (orange). Nucleic acids were stained with DAPI. **a** Overview of the biopsy, overlay of all fluorescence channels and background fluorescence allows orientation within the tissue (**b**). At higher magnification of the inset marked in (**a**), single rods are located at the border of the dermis and the subcutaneous fat tissue. **c** Inset of (**b**) showed the detected rods at higher magnification (**d** to **g**), the identical microscopic field as (**c**) with separate fluorescence channels, DAPI, Cy3, Cy5, and FITC, respectively. Note the detected rod was positive in DAPI (**c**), the PRAV (**d**), and EUB338 (**e**) probe, but showed no signal with PAC (**f**)

**Fig. 3:**
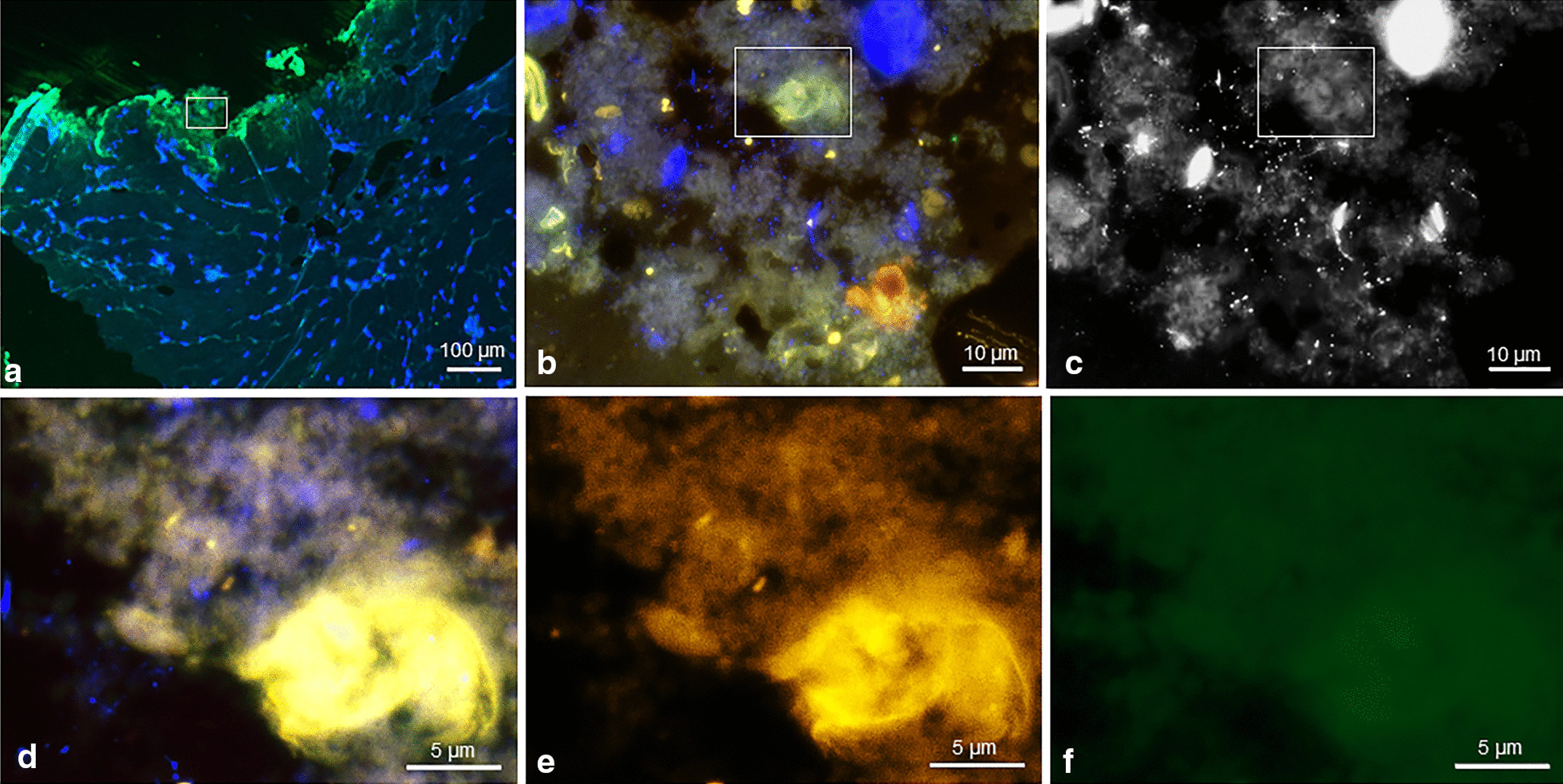
FISH of a *C. avidum* culture positive tissue (capsule tissue) biopsy fixed during surgery, shows single *C. avidum* cells in the tissue. The tissue section was hybridized with the panbacterial probe EUB338_Cy5_ (mangenta), the *C. acnes/C. modestum/C. namnetense*-specific probe PAC_FITC_ (green) and the *C. avium/ A. thiooxidans, A. propionicum, A. jensenii, A. thoeni*-specific probe PRAV_Cy3_ (orange). Nucleic acids were stained with DAPI. **a** Overview of the biopsy, overlay of all fluorescence channels and background fluorescence shows the destruction of the tissue, also visible at higher magnification of the inset (**b**) marked in (**a**). **c** The identical microscopic field in the DAPI channel only (black and white) shows multiple DAPI-positive pleomorphic microorganisms scattered in the tissue. **d** Inset of (**c**) at higher magnification single rods are visualized in the tissue. **e** Some rods stained positive with PRAV in the Cy3 channel but no signal was detected with the PAC in the FITC channel (**f**)

In all samples, few single bacteria were detected by nucleic acid stain DAPI only, whereas EUB338, PRAV and PAC probe were negative. These single bacteria are visualized according to size and morphology and frequently located in the subcutaneous fat tissue. In one case, we detected FISH positive bacteria, indicating ribosome-rich and therefore presumably active bacteria [29–32]. Using the specific *C. avidum/ A. acidipropionici/ A. propionicum/ A. jensenii/ A. thoeni*- probe, we did not find any FISH-positive bacteria in the lumen of the sweat glands. However, we did find bacteria positive with the PRAV probe close to structures compatible with sweat glands, located at the interface between dermis and subcutaneous fat tissue (Fig. 2). One positive control with culture *C. avidum* positivity (pseudocapsule tissue biopsy from an infected arthroplasty with *C. avidum)* was analyzed for comparison with the dermis of uninfected cases and revealed a higher number of bacteria. Among DAPI-positive cells single FISH-positive bacteria were detected by the EUB3338 and PRAV probe (Fig. 3).

## Discussion

Prevention of PJI is key to avoid re-operations and prolonged antibiotic treatment after arthroplasty surgery [33]. One of the current prevention strategies is immediate skin antisepsis before surgery in conjunction with single dose antibiotic prophylaxis 30–60 minutes prior to surgery to eliminate viable bacteria on the skin, which we also used in this study. *C. avidum* is a natural inhabitant of the human skin in particular in obese patients [10, 11] and recently recognized as a relevant pathogen in hip PJIs [7–9]. In our study of 60 patients, 12 (20%) were colonized with *C. avidum* prior to hip surgery. Pre-operative skin antisepsis eliminated *C. avidum* in 80% of patients. In fact, we found viable *C. avidum* in 20% of the patients on the skin surface, which might be a risk factor for intraoperative colonization of the joint prosthesis and thus for developing a PJI. We strictly followed the required exposure time of antiseptics given by the manufacturer specifications (one minute exposure time before major interventions) and by the in-house guidelines of a minimum of three antiseptical rounds. This may not be enough when implanting foreign material.

This is the first study revealing the insufficient antisepsis of *C. avidum* on the skin. Other studies focusing on different microorganisms and different skin antisepsis strategies show similar results [8, 34, 35]. In a randomized trial, Patrick et al. showed that skin antisepsis with sequential application of PVI and CHG reduced the bacterial numbers in the surgical wound more effectively than PVI alone in patients undergoing spinal surgery [8]. Blonna et al. found similar results focusing on coagulase-negative staphylococci and *C. acnes* [35]. For *C. acnes*, a recent randomized study revealed a higher reduction of *C. acnes* after skin preparation with topical benzoyl peroxide than with chlorhexidine [36]. Heckmann et al. evaluated whether higher concentrations of chlorhexidine with an additional mechanical scrub would be more effective in eliminating growth of *C. acnes* than standard antisepsis [37]. No significant difference was found leading to the conclusion that also mechanical scrub cannot eradicate *C. acnes* in deeper layers of the skin. Further studies are needed to improve antisepsis strategies of the skin prior to surgery with focus on reaching the dermis including the subepidermal glands for completely killing any viable bacteria on the skin at time of surgery. For that, an innovative approach could be the application of photodynamic therapy (PDT) [34, 38–41] to reduce colonizing bacteria. In a recent study we showed a 100% reduction of viable bacteria after skin antisepsis and PDT with methyl aminolevulinate (MAL) [34]. The treatment, however, led to transient skin erythema which is an obstacle for immediate surgery. PDT is certainly very promising but the optimal parameters with the ideal balance of bactericidal effect versus skin irritation have to be investigated.

In one patient, we visualized bacteria at the border of the subcutaneous fat tissue indicating a possible niche for bacteria protected from skin antisepsis and thus a potential source for contamination of deep peri-implant tissue at time of surgical incision and implantation of the hip prosthesis. A recent study reported the presence of bacteria in deep tissues, which were considered as sterile areas [42], leading to the assumption that not only the superficial skin bacteria but also bacteria in the dermis may find the way to deeper structures and infect an implant. Lee et al. found viable *C. acnes*—another skin commensal—in the dermal tissue in 7 out of 10 male volunteers after surface skin antisepsis [43]. Remaining bacteria in dermal glands can be a source of inoculation of deeper structures by surgical incision [44]. There is an increasing number of studies describing *C. acnes* remaining in dermis despite skin antisepsis [45, 46]. However, we found no viable bacteria in the dermis with routine tissue culture methods. No other study than ours exists so far focusing on *C. avidum* persistance on skin surface and/or in deeper structures after surgical antisepsis*.* In our study, the FISH-results showed, that in most of the cases, only a few single bacteria per section were detected, mostly in the subcutis stained by DAPI, but the FISH probes showed no signal, which indicate a rather resting or inactive state. Only in one case, DAPI-positive bacteria were also tested FISH-positive indicating ribsosome-rich and presumably viable *C. avidum*. The low numbers of FISH-positive bacteria, however, could be influenced by the routine single antibiotic prophylaxis 30–60 min before the start of the surgery aiming bactericidal antibiotic concentration in the operating field.

A limitation of our study is the intraoperative focus on *C. avidum* and not on other colonizing bacteria, which resulted in evaluating the skin antisepsis effect in a low number of patients. Another limitation is the fact that we did not inhibit the antiseptics and possibly transferred traces into the eSwabs during the scraping of the skin. This might have caused inhibition and an underestimation of the actual bacterial growth. Since we focused on *C. avidum*, which has a prolonged cultivation time, screening for skin colonization had to be completed 7–14 days before surgery and may be different than a screening performed on the day of surgery.

## Conclusion

Standard skin antisepsis with povidone-iodine/alcohol in combination with antibiotic prophylaxis incompletely eliminates *C. avidum* from the groin area at time of surgical incision of the skin. With the one positive *C. avidum* FISH result, we can only illuminate but not prove our hypothesis that *C. avidum* in the dermis might be a potential source for infecting prostheses perioperatively. The clinical significance of our study is to highlight the orthopedic surgeon’s diligence regarding the treatment of wound margins with care, especially when implanting prosthetic material, since we showed that the skin antisepsis is not efficient in eliminating all colonizing bacteria. Future studies are needed to find skin antisepsis with more potency or other antisepsis methods reaching the deeper tissue of the dermis.

## Supplementary information

**Additional file 1.** Supplement.

## Data Availability

All data generated or analysed during this study are included in this published article and its supplementary information files.

## Abbreviations

PJI: Periprosthetic joint infections
THA: Total hip arthroplasty
FISH: Fluorescence in situ hybridization
PVI: Alcoholic povidone-iodine
CHG: Chlorhexidine gluconate
CNS: Coagulase-negative staphylococci
