# Supplementary material for: Cutibacterium avidum resists surgical skin antisepsis in the groin—a potential risk factor for periprosthetic joint infection: a quality control study
Source: Antimicrob Resist Infect Control. 2021 Feb 1;10:27. doi: 10.1186/s13756-021-00883-1 (PMC7852298; doi:10.1186/s13756-021-00883-1)
Supplement: Supplementary file 1 — Additional file 1. Supplement. [file 13756_2021_883_MOESM1_ESM.docx]

**Supplement**

Table S1: Basic characteristics of the 60 enrolled patients with a screening skin swab

| Characteristic | No. (%) |
| --- | --- |
| Age – year, median [range] | 67 [40-87] |
| Female sex | 28 (46.7) |
| BMI - kg/m^2^  All  Median [range]  *C. avidum*-positive  Median [range] | 26.7 [19.1-39.1]  32.1 [21.8-39.1] |
| Underlying joint disorder for primary arthroplasty  Osteoarthritis  Femoral head necrosis | 59 (98.3)  1 (1.7) |
| Hip side  left  right | 33 (55)  27 (45) |
| Microbiome analysis at screening  *Cutibacterium* sp.  *C. avidum*  *C. acnes*  *C. granulosum*  Coagulase-negative staphylococci  *S. epidermidis*  *S. hominis*  *S. capitis*  *S. haemolyticus*  *S. warneri*  *S. pettenkoferi*  *S. lugdunensis*  *S. simulans*  *Enterococci sp.*  *E. faecalis*  *E. faecium*  *Staphylococcus aureus*  *Corynebacterium sp.*  *C. tuberculostearicum*  *C. glucuronolyticum*  *C. singular*  *C. accolens* | **23 (38.3)**  **12 (20)**  11 (18.3)  2 (3.3)  **47 (78.3)**  29 (48.3)  22 (36.7)  8 (13.3)  6 (10)  3 (5)  2 (3.3)  2 (3.3)  1 (1.7)  **2 (3.3)**  1 (1.7)  1 (1.7)  **2 (3.3)**  **4 (6.7)**  1 (1.7)  1 (1.7)  1 (1.7)  1 (1.7) |

**Evaluation of the specific probe PRAV for the detection of *Cutibacterium avidum* by FISH.**

**Table S2: In silico evaluation of the newly designed *Cutibacterium avidum* specific probe PRAV using the software probe check** (<http://131.130.66.200/cgibin/probecheck/content.pl?id=home>).

| Probe | Sequence orientation | No. of mis-matches to probe | Target species | Genbank  No. |
| --- | --- | --- | --- | --- |
| PRAV | 5´ GCC CCA AGT TTT CAC TTC CG 3` | 0 | ***Cutibacterium avidum***  *Acidipropionibacterium acidipropionici*  *Acidipropionibacterium propionicum*  *Acidipropionibacterium jensenii*  *Acidipropionibacterium theonii* | AJ003055  AJ704570  AJ003058  AJ704571  AJ704572 |
|  | 5´ GCC CCA AGA TTT CAC TTC CG 3`  5´ GCC CCG AGT TTT CAC TTC CG 3` | 1 | *Acidipropionibacterium microaerophilum*  *Micropruina glycogenica* | AF234623  AB012607 |
|  | 5´ GCC CCA AGA TTA CAC TTC CG 3` | 2 | ***Cutibacterium acnes***  and others | AB042288 |

*Acidipropionibacterium acidipropionici, Acidipropionibacterium jensenii,* and *Acidipropionibacterium theonii as well as Acidipropionibacterium microaerophilum* and *Micropruina glycogenica* are phylogenetically closely related to *C. avidum (1-3)*. They are clinically not relevant and primarily used in biotechnology productions. *Acidipropionibacterium acidipropionic and Acidipropionibacterium propionicum* are part of the human skin microbiome(4)*. Acidipropionibacterium propionicum* has been rarely detected in oral and eye infections only (5-7).

**Table S3: In silico evaluation of the specific probe PAC using the database silva**

| Probe | Sequence orientation | No of mis-matches to probe | Target species | Genbank  No. |
| --- | --- | --- | --- | --- |
| PAC | 5´ GCC CCA AGA TTA CAC TTC CG ` | 0 | ***Cutibacterium acnes,***  *Cutibacterium modestum,*  *Cutibacterium namnetense*  *Acidithiobacillus thiooxidans*  *Mycobacterium chitae* | AB042288  AFAM01000003  AFUN01000008  AF36021  M29560 |
|  | 5´ GCC CCA AGA TTT CAC TTC CG 3` | 1 | *Acidipropionibacterium microaerophilum* | AF234623 |
|  | 5´ GCC CCA AGT TTT CAC TTC CG 3` | 2 | ***Cutibacterium avidum***  and other | AJ003055 |

The probe PAC shows 100% homology to *Cutibacterium modestum,* which was originally isolated from the humeral membrane of a patient who underwent revision of a failed total shoulder arthroplasty (8). *Cutibacterium namnetense* was published in the context of bone infections (9). *Acidithiobacillus thiooxidans* and *Mycobacterium chitae* are clinically not relevant in PJIs. *Acidithiobacillus thiooxidans* was first isolated from compost oil and is commonly used in metal extraction (10, 11). *Mycobacterium chitae* was isolated from soil by chicken passage (12, 13) and is rarely published as cause of tuberculosis.

**Table S4:** Specificity Test of probe PAC and PRAV against typical skin flora species and clinically relevant species using the hybridization conditions optimal for the PRAV-probe.

| **Strain** | **Probe PRAV** | **Probe PAC** | **Probe EUB338** |
| --- | --- | --- | --- |
| *Cutibacterium avidum* | positive | negative | positive |
| *Cutibacterium acnes* | negative | positive | positive |
| *Staphylococcus epidermidis* ATC1228 | negative | negative | positive |
| *Staphylocccus aureus*  ATCC29213 | negative | negative | positive |
| *Enterococccus faecalis*  ATCC29212 | negative | negative | positive |
| *Enterococcus faecium*  (MB18206) | negative | negative | positive |
| *Streptococcus pyogenes*  Clinical isolate | negative | negative | positive |
| *Streptococcus pneumoniae*  ATCC6031 | negative | negative | positive |
| *Lactococcus lactis*  ATCC 19435 | negative | negative | positive |
| *Micrococcus luteus* MI3307908 | negative | negative | positive |
| *Corynebacterium mucifaciens* DSM44265 | negative | negative | positive |
| *Corynebacterium matruchotii*  DSM 20635 | negative | negative | positive |
| *Fructilactobacillus florum*  JCM 16035 | negative | negative | positive |
| *Bacillus cereus*  1306 | negative | negative | Positive |
| *Escherichia coli*  ATCC90028 | negative | negative | positive |
| *Escherichia coli*  ATCC25922 | negative | negative | positive |
| *Pseudomonas aeruginosa*  MV26117 | negative | negative | positive |
| *Candida albicans*  ATC90028 | negative | negative | Positive |
| *Aspergillus fumigatus*  ATCC9197 | negative | negative | positive |

**Figures S1.** Evaluation of stringency by optimization of hybridization conditions for probe PRAV differentiates *C. avidum* and *C. acnes* at formamide concentrations between 0 and 20% with an optimum at 10% formamide.

Hybridization buffers with different formamide concentrations were tested to define optimal stringency conditions for probe PRAV. Therefore, FISH was applied on fixed *C. acnes* and *C. avidum* cells with formamide concentrations varying from 0 to 70% in the hybridization mix. Signal intensity (relative fluorescent Units RU) was determined using the software (Zen 2, module image processing, Carl Zeiss, Jena). Images were taken with a fixed exposure time and minimum of 10 images (minimum of 1000 cells) per formamide concentration. PRAV clearly differentiated the two strains at formamide concentrations between 0 and 20% with an optimum at 10% formamide. PRAV clearly differentiates the two strains at formamide concentrations between 0 and 20% with an optimum at 10% formamide.

**Figure S2: Probe PRAV specifically detects *C. avidum* at 10% formamide concentration.** Hybridization of fixed cells of *C. avidum* (A and C) and *C. acnes* (B and D) was performed with probes EUB338-Cy5 (mangenta) and PRAV-Cy3 (orange) at 10% formamide. Whereas *C. avidum* was detected by EUB338 (A) and PRAV (C) in identical microscopic fields, *C. acnes* was detected by EUB338 only (B, D).


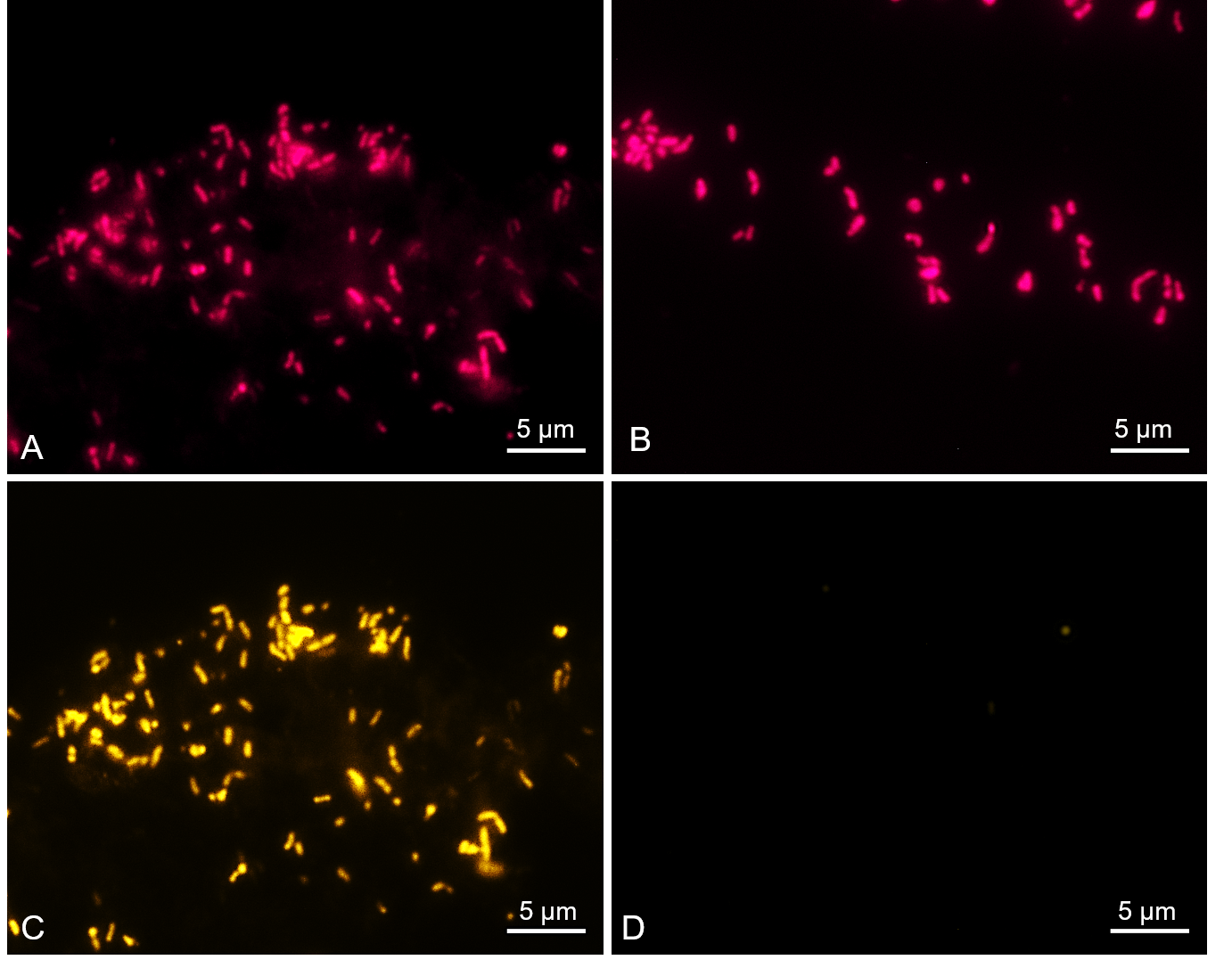


**References**

1. Guan N, Du B, Li J, Shin HD, Chen RR, Du G, et al. Comparative genomics and transcriptomics analysis-guided metabolic engineering of *Propionibacterium acidipropionici* for improved propionic acid production. Biotechnol Bioeng. 2018;115(2):483-94.

2. Liu L, Guan N, Zhu G, Li J, Shin HD, Du G, et al. Pathway engineering of *Propionibacterium jensenii* for improved production of propionic acid. Sci Rep. 2016;6:19963.

3. Paik HD GB. Enhanced bacteriocin production by *Propionibacterium thoenii* in fed-batch fermentation. J Food Prot. 1997;60(12):1529-33.

4. Khayyira AS RA, Irianti MI, Malik A. Simultaneous profiling and cultivation of the skin microbiome of healthy young adult skin for the development of therapeutic agents. Heliyon. 2020:6(4):e03700.

5. Siqueira JF RI. Polymerase chain reaction detection of *Propionibacterium propionicus* and *Actinomyces radicidentis* in primary and persistent endodontic infections. Oral Surg Oral Med Oral Pathol Oral Radiol Endod. 2003;96(2):215-22.

6. Brazier JS HV. *Propionibacterium propionicum* and Infections of the Lacrimal Apparatus. 1993;17(5):892-3.

7. Chávez De Paz LE MA, Dahlén G. Gram-positive rods prevailing in teeth with apical periodontitis undergoing root canal treatment. Int Endod J. 2004;37(9):579-87.

8. Butler-Wu SM, Sengupta DJ, Kittichotirat W, Matsen FA, 3rd, Bumgarner RE. Genome sequence of a novel species, *Propionibacterium humerusii*. J Bacteriol. 2011;193(14):3678.

9. Corvec S GA, Aubin GG, et al. Rifampin-Resistant *Cutibacterium* (formerly *Propionibacterium*) *namnetense* Superinfection after *Staphylococcus aureus* Bone Infection Treatment. J Bone Jt Infect. 2018;3(5):255-7.

10. Waksman SA JJ. Acid production by a new sulfur-oxidizing bacterium. Science. 1921;53(1366):216.

11. Yang L ZD, Yang J, Wang W, Chen P, Zhang S, Yan L. *Acidithiobacillus thiooxidans* and its potential application. Appl Microbiol Biotechnol. 2019;103(19):7819-33.

12. Tsukamura M. *Mycobacterium chitae*: a new species. Jpn J Microbiol. 1967;11(1):43-7.

13. Chwalibóg B JM, Maliszewska Z, Michalowska D, Zbikowski H. A case of tuberculosis caused by atypical mycobacteria identified as *Mycobacterium chitae*. Gruzlica. 1971;39(1):57-60.
